# High-Performance Metal-Supported Protonic Ceramic Fuel Cell Utilizing Ammonia-Derived Fuel

**DOI:** 10.1021/acsami.5c18211

**Published:** 2026-05-19

**Authors:** Xuemei Li, Hanchen Tian, Bo Guan, Qingyuan Li, Lingfeng Zhou, Awa Kalu, Shaoshuai Chen, Xinyuan Zhu, Yu Xie, Siyuan Liu, Xingbo Liu, Wenyuan Li

**Affiliations:** † Chemical and Biomedical Engineering Department, Benjamin M. Statler College of Engineering Mineral Resources, 5631 West Virginia University, Morgantown, West Virginia 26506, United States; ‡ Mechanical Aerospace Engineering Department, Benjamin M. Statler College of Engineering Mineral Resources, 5631 West Virginia University, Morgantown, West Virginia 26506, United States; § DOE National Energy Technology Laboratory, 3610 Collins Ferry Road, Morgantown, West Virginia 26505, United States

**Keywords:** protonic ceramic fuel cells, metal-supported PCFC, ammonia fuel, cosintering, proton-conducting electrolyte

## Abstract

Metal-supported protonic ceramic fuel cells (MS-PCFCs) are attractive for ammonia-based energy conversion, but their development is limited by coupled challenges associated with cosintering, fuel electrode reduction, and chemical stability under NH_3_. In this work, these issues are examined using a Ni metal-supported PCFC as a representative system. By correlating reduction conditions and electrolyte processing, a fabrication window is identified that enables cosintering of a dense BaZr_0.1_Ce_0.7_Y_0.1_Yb_0.1_O_3−δ_ (BZCYYb1711) electrolyte with a porous Ni support while maintaining structural integrity. The incorporation of an anode functional layer improves interfacial compatibility and reduces ohmic resistance without disrupting electronic continuity. The interplay among electrolyte composition, sintering temperature, and Ni diffusion is further clarified, highlighting the trade-off between densification and protonic conductivity. For ammonia operation, an upstream NH_3_ predecomposition strategy is employed to reduce chemical stress on the anode during long-term testing. Under these conditions, the MS-PCFC achieves peak power densities of 0.72 W cm^–2^ with NH_3_ and 1.05 W cm^–2^ with H_2_ at 700 °C and sustains stable operation for 300 h under NH_3_ at 600 °C. The results provide practical insight into processing and microstructural considerations for Ni-based MS-PCFCs operated with ammonia.

## Graphical abstract

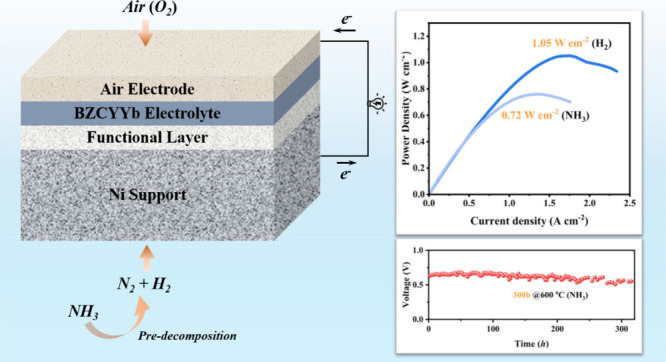

## 1 Introduction

Solid oxide fuel cells (SOFCs) have consistently attracted attention as ideal energy conversion devices, noted for their high efficiency in converting chemical energy into electrical power, while offering low emissions and flexibility across different fuel types.^1,2^ Metal-supported solid oxide fuel cells (MS-SOFCs) are characterized by thin layers of electrochemically active ceramics supported on thicker metal layers that provide mechanical support and electronic current collection.^3^ MS-SOFCs have recently experienced noteworthy progress, marked by cost reductions, enhanced mechanical strength, increased tolerance to redox cycles, and improved ability to withstand repeated and rapid thermal cycles.^4–8^ These advancements have garnered a significant amount of attention because of the potential of MS-SOFCs in efficient energy conversion. Despite these advances, the development of metal-supported proton-conducting fuel cells (MS-PCFCs), which operate at lower temperatures, offer higher efficiency, exhibit slower aging, and enable reactions to occur at the cathode side, thus preventing a reduction in H_2_ partial pressure at the anode side, has been relatively limited. A major obstacle hindering the development of MS-PCFCs lies in the intricate anode substrate fabrication process required to achieve both high and stable operational performance. The compatibility of ferritic steel with ceramics, coupled with their similar thermal expansion coefficients (TECs) (10–12 ppm/K), positions it as an excellent candidate for anode support.^9^ However, Mercadelli et al. introduced stainless steel support into proton-conducting anodes BZCY-Ni and observed a significant issue of contamination of the Ni catalyst and melting of the stainless steel due to the diffusion of iron, chromium, and nickel between ferritic FeCr steel and Ni-containing anodes.^10^ Besides, a substantial amount of Si, along with a minor proportion of Cr, would migrate from the stainless steel into the cell components, highlighting the imperative for employing more complex and demanding thin-film deposition and post-treatment procedures.^11^

Among metallic materials, Ni holds a unique position in SOFCs due to its superior electrochemical activity.^12^ Ni exhibits high conductivity to the H_2_ oxidation reaction compared to other metals such as Co, Fe, Pt, Mn, and Ru.^13,14^ Nevertheless, when Ni is used as the metal support, overcoming the challenge of ensuring the sintering of a dense electrolyte on the Ni metal is still a critical aspect that necessitated a thorough exploration of fabrication methodologies. Typically, achieving optimal dense bulks/films for BaCeO_3_-based and BaZrO_3_-based perovskite materials requires sintering temperatures exceeding 1500 °C, leading to potential undesirable reactions with the supporting electrode materials, element evaporation, or undesired phase formation.^15–19^ Researchers have studied the optimization of fabrication parameters, including sintering temperature, sintering aids, and composition, to strike the delicate balance that is essential for stable SOFC operation.^20,21^ However, this remains an uncharted territory for the field of MS-PCFCs.^22–24^

When it comes to fuel, ammonia has emerged as a promising hydrogen carrier because it can be stored and transported under ambient pressure and decomposes into hydrogen at temperatures below the operating range of PCECs.^25–27^ Ammonia decomposition follows a well-established reaction pathway, which can be described as a sequence of elementary dehydrogenation steps. (i) Ammonia is adsorbed on the active sites of the catalyst. (ii) The N–H bond is successively cleaved to form H atoms. (iii) N and H atoms desorb from the active site to form N_2_ and H_2_ gas.^28^ Based on density functional theory, it has been demonstrated that the rate-limiting steps in this reaction sequence are generally associated with N–H bond cleavage and N–N recombination, and their relative significance depends strongly on the catalyst surface.^29,30^ Catalytic promotion strategies, such as transition metal infiltration, noble metal incorporation, or the use of high-entropy alloys, are understood to primarily reduce the activation barrier for N–H bond scission and to facilitate N_2_ desorption. Xiong et al. demonstrated that Ba- or Co-modified Ni systems improved decomposition rates, with Ba-Ni/YSZ achieving 100% NH_3_ conversion at 600 °C and Co alloying simultaneously boosting catalytic activity and electronic conductivity.^31^ Beyond Ni-based alloys, noble metal incorporation provided another pathway to accelerate ammonia decomposition. The Pd-infiltrated BZCY reached 580 mW cm^–2^ at 600 °C, while the Ni-BZCYYbPd reached 724 mW cm^–2^ at 650 °C with complete NH_3_ conversion.^32^ In parallel, structural innovations such as in situ exsolution were developed, allowing Ni or Pd nanoparticles to emerge dynamically from perovskite lattices, thereby forming highly active and stable catalytic sites with strong resistance to nitridation. The stability of high-temperature PCFCs under ammonia is a prerequisite for their practical application, yet cells based on conventional electrode materials often suffer pronounced performance degradation. Among the various degradation factors, processes at the fuel electrode are particularly critical. Ni in conventional fuel electrodes is prone to nitridation in the presence of ammonia, forming nickel nitrides (Ni_*x*_N_*y*_) or NO_*x*_ species, which decreases catalytic activity, increases anode resistance, and accelerates performance decay.^26^ Since SOFC and PCFC systems are expected to operate for tens of thousands of hours, the continued interaction between Ni and ammonia inevitably leads to progressive nitridation of the Ni substrate, thereby compromising the mechanical and electrochemical stability of the electrode over long-term operation. This concern is intrinsic to Ni-based fuel electrodes, as the nitridation process can persist as long as ammonia or ammonia-derived species are present at the anode.

The present study delves into the intricacies of the fabrication process of MS-PCFCs using Ni as support, encompassing the anode functional layer (AFL), anode reduction, and anode–electrolyte cosintering processes, to optimize stability and compatibility with the electrolyte. Furthermore, the study introduces a practical approach to address limitations associated with NH_3_ fuel utilization in MS-PCFC systems by introducing a highly efficient catalyst into the gas flow tube channel to create a functional reaction zone for NH_3_ predecomposition. A performance of 0.48 W/cm^2^ at 650 °C was achieved with NH_3_, which is comparable to H_2_-fed performance. During a 300 h operation demonstration, the MS-PCFC showed relatively good stability. This study could contribute significantly to the growing body of knowledge in the field, pushing the boundaries of MS-PCFC technology toward a cleaner and more sustainable energy future.

## 2 Experimental Section

### 2.1 Synthesis of Materials

The perovskite BaZr_0.1_Ce_0.7_Y_0.1_Yb_0.1_O_3−δ_ (BZCYYb1711) electrolyte was synthesized by the sol–gel method in which stoichiometric Ba­(NO_3_)_2_, Ce­(NO_3_)_3_·6H_2_O, Zr­(NO_3_)_3_·6–7H_2_O, Y­(NO_3_)_3_·6H_2_O, and Yb­(NO_3_)_3_·5H_2_O were first dissolved in distilled water. Then, complex reagents EDTA and citric acid (CA) were sequentially added to the solution in a 1:1:1.25 total metal ion:EDTA:CA mole ratio. The pH of the solution was adjusted to 8–10 using aqueous ammonia. The solution was heated to 80 °C until a viscous gel formed, which was pretreated at 530 °C for 5 h and then calcined at 1150 °C for 10 h under an air atmosphere to obtain BZCYYb1711 powders.

Ruddlesden–Popper (RP) phase cathode material Pr_2_NiO_4+δ_ (PNO) was synthesized by a sol–gel method in which Pr­(NO_3_)_3_·6H_2_O and Ni­(NO_3_)_2_·6H_2_O were first dissolved in distilled water. Then, complex reagents EDTA and CA were sequentially added to the solution in a 1:1:1.25 total metal ion:EDTA:CA mole ratio. The pH of the solution was adjusted to 6–8 using aqueous ammonia. The solution was heated at 80 °C until a viscous gel formed, which was pretreated at 500 °C for 5 h and then calcined at 1150 °C for 10 h under an air atmosphere to afford PNO powders.

### 2.2 Fabrication of the Anode-Supported Single Cell

Anode-supported cells with a configuration of NiO||NiO-BZCYYb||BZCYYb||PNO were fabricated by the following procedure. First, fine nickel oxide (NiO-F, 3–4 m^2^/g, Nexceris, LLC) powder, coarse nickel oxide (NiO-S, <1 m^2^/g, Nexceris, LLC), polyvinyl butyral (PVB), and starch as the pore former with a weight ratio of 40:60:4.5:10 in ethanol were milled for 1 h. After drying, 0.50 g of anode powder was pressed for 20 s under 7000 lb using a 15 mm diameter steel mold. Next, the AFL powder was prepared by mixing 60 wt % fine NiO powder and 40 wt % BZCYYb powder and then ball-milled for 3 h. After drying, 3 g of AFL powders was mixed with 4 mL of ink vehicle (Nexceris, LLC). The powder/ink mixture was ground for 1 h to yield the resultant paste as an AFL slurry. The BZCYYb electrolyte slurry and cathode slurry were prepared in the same manner. The AFL slurry and electrolyte slurry were spin-coated sequentially onto the NiO layer, with each layer applied twice at a rotational speed of 7800 rpm for 20 s per coating, followed by drying in an oven at 80 °C for 1 h. The pellets were then pressed at 20 000 lb for 20 s to form half-cells. The half-cell was sintered at 1350 °C for 5.5 h to densify the BZCYYb electrolyte, using a heating and cooling rate of 3.5 °C min^–1^ down to room temperature. The cathode slurry was brush printed onto the half-cell and followed by calcining for 2 h at 1000 °C. Silver wires were fixed on the anode and cathode surfaces using silver paste (Col-int Tech). Afterward, the single cell was sealed to the top of the aluminum tube using ceramabond 552 (Aremco Inc.) high-temperature adhesive.

In order to evaluate the conductivity of the electrolyte, BZCYYb powder was mixed with PVB (9% solution prepared in advance using ethanol as a solvent) at a weight ratio of 10:1. This mixture was manually milled in a mortar, ensuring thorough blending. After the initial mixing and subsequent drying, the resulting material was once again ground into a fine powder using a mortar. Subsequently, 1.0 g of the powder was pressed into pellet using a 15 mm mold under 10 000 lb. The pressed electrolyte pellets were then positioned in a high-temperature furnace and calcined at 1350 °C for a duration of 5.5 h. Silver paste was applied to both sides of the sintered BZCYYb pellets with an area of 0.31 cm^2^, and four silver wires were attached to the two sides of Ag paste.

### 2.3 Electrochemical Measurement and Characterization

The electrochemical performances of single cells were tested in a four-probe mode by a Gamry Interface 5000E instrument. The cells were sealed to the top of an alumina tube with the anode exposed to fuel and the cathode to the ambient air. The assembled cells were placed in a horizontal tube furnace. H_2_ was used to reduce the NiO in both the anode and the anode functional layer at 600 °C for 1.5 h; 100 sccm of air was simultaneously supplied to the air electrode side, and the open-circuit voltage (OCV) was continuously monitored in real time. The detailed reduction procedure is included in section 3.1. The performances were tested after the OCV stabilized. The current–voltage–power curves were collected from OCV to 0.4 V from 600 to 700 °C. Impedance spectroscopy at OCV for the full cell was carried out with variable frequency from 106 to 0.01 Hz from 600 to 700 °C. In the ammonia durability test, 100 sccm of H_2_ was fed into the anode side first and switched to ammonia (70 sccm) after performance evaluation under H_2_. For the conductivity measurement, silver paste was applied to both sides of the sintered BZCYYb pellets with an area of 0.31 cm^2^, and four silver wires were attached to the two sides of the Ag paste. Electrochemical impedance spectroscopy (EIS) was then carried out with variable frequency from 10^6^ to 0.01 Hz under a 40 vol % H_2_O humidified air atmosphere from 600 to 450 °C, and the electrical conductivity was calculated from the EIS results. To evaluate the redox tolerance of the porous Ni metal substrate, a single redox cycle was carried out on a previously tested cell. After the initial reduction and electrochemical characterization at 700 °C under standard fuel/air conditions, the fuel electrode gas was switched to 1% O_2_ in N_2_ at 700 °C and maintained for 2 h to reoxidize the Ni substrate. Air was then fed to both electrodes to confirm complete reoxidation by the disappearance of the OCV. Subsequently, the fuel electrode was switched back to the fuel gas, and the cell was rereduced under the same conditions as the initial activation, followed by EIS and *I–V* measurements.

The *H*/*D* ratio (maximum deflection height/diameter) was determined on the basis of direct dimensional measurements using a vernier caliper with an accuracy of 0.01 mm. For each sample, the diameter was measured at three different positions, and the average value was used as the representative diameter. The maximum deflection height was measured at the highest point on the curved surface relative to the base plane. The *H*/*D* values were then calculated from the average diameter and measured height.

The phase purity was examined by X-ray diffraction (XRD, PANalytical X’pert PRO, Cu Kα radiation). The microstructural and compositional properties of the samples were examined by scanning electron microscopy (SEM, Hitachi S-4700) and scanning transmission electron microscopy (STEM). STEM was performed using a Themis Z system (Thermo Fisher) equipped with energy dispersive spectroscopy (EDS) and operated at an acceleration voltage of 200 kV. EDS mapping by STEM was performed with drift correction, dwell times of 50–100 μs, and at least 60 frames acquired for each location.

## 3 Results and Discussion

### 3.1 Structural and Electrochemical Influence of the Support

#### 3.1.1 Support/Electrolyte Sintering

In contrast to the conventional cermet support, the Ni metal support sintering process exhibits a distinct shrinkage, potentially detrimental to the full cell’s structure if it is not properly managed. To better understand the sintering compatibility between the NiO-based metal support and the BZCYYb electrolyte, we investigated the shrinkage behavior of supports prepared using different ratios of NiO-F and NiO-S powders (Figure S1). To quantitatively evaluate the shrinkage compatibility, the curvature of the cosintered half-cells was analyzed using the *H*/*D* ratio (Table S1). The *H*/*D* value decreases progressively with an increase in NiO-S content and reaches a minimum (∼0.054) at S:F = 3:2, indicating the most balanced densification behavior between the support and electrolyte. To elucidate the effect of particle size tailoring on the shrinkage behavior of the support, the morphology and particle size distributions of the NiO powders are presented in Figure S2. NiO-F exhibits a narrow submicrometer distribution (∼0.1–0.3 μm), NiO-S a markedly coarser one (∼0.8–1.0 μm), while the mixed powder (S:F = 3:2) a clear bimodal profile (∼0.25 and ∼0.7 μm). Such bimodality provides a rigid skeleton of coarse particles and interstitial filling by fines, which balances packing density and sintering kinetics, thereby reducing densification-driven shrinkage and warping during cosintering.

#### 3.1.2 Reduction of Support

We investigated the NiO to Ni reduction procedure in the metal support to avoid catastrophic failure. Low and high H_2_ concentrations were used for the reduction. The Ni metal support showed significantly greater shrinkage under high H_2_ concentrations compared to the cermet anode (Table S2), while a lower H_2_ concentration effectively mitigated the shrinkage of the Ni-supported anode. The slight increase in diameter after reduction is likely associated with the release of tensile stress in the electrolyte layer of the as-prepared sample, which bends the anode substrate back upon reduction.^33^ To provide a more reliable assessment of the structural evolution, the porosity of reduced Ni and cermet bars was further measured by using the Archimedes method. The porosity of pure Ni increases from 26% under high H_2_ concentrations to 34% under low H_2_ concentrations. SEM observations reveal that reduction under low H_2_ concentrations produces Ni with fine and abundant pores within the particles (Figure 1b,e), whereas reduction under high H_2_ concentrations results in fewer and larger pores (Figure 1a). Under high-H_2_ conditions, the Ni network undergoes coarsening and coalescence, as evidenced by the reduced overall sample dimensions, leading to decreased porosity. In contrast, the cermet samples exhibit nearly unchanged porosity (41% vs 42%) regardless of the H_2_ concentration used (Figure 1c,d). This is because the rigidity provided by BZCYYb in the cermet helps retain the overall dimensions of the sample during reduction. Moreover, the dwell time under a low hydrogen concentration also proves to be crucial for maintaining the stability and microstructure of the cell. We examined rapid versus slow reduction processes on OCV stability (Figure S3). During rapid anode reduction, 5% H_2_ balanced with N_2_ was initially introduced to the anode, stabilizing the OCV after 5 min. The H_2_ concentration was then increased to 10% and 20% at 5 and 10 min intervals, respectively, resulting in significant fluctuations in the OCV curve and severe cracking of the cell (Figure S3b). Conversely, during a slow reduction, 10% H_2_ balanced by N_2_ was initially supplied and maintained for approximately 1.5 h. The OCV steadily increased to 0.9 V after 30 min and reached 0.95 V at 700 °C as the H_2_ content was gradually increased to 100%. The slow reduction process stabilizes the microstructure and avoids leakage of the electrolyte. These findings highlight the difference between conventional cermet and the metal-supported cells. It is important to optimize the reduction procedure for MS-PCFC, ensuring cell stability and high cell performance.

**Figure 1:**
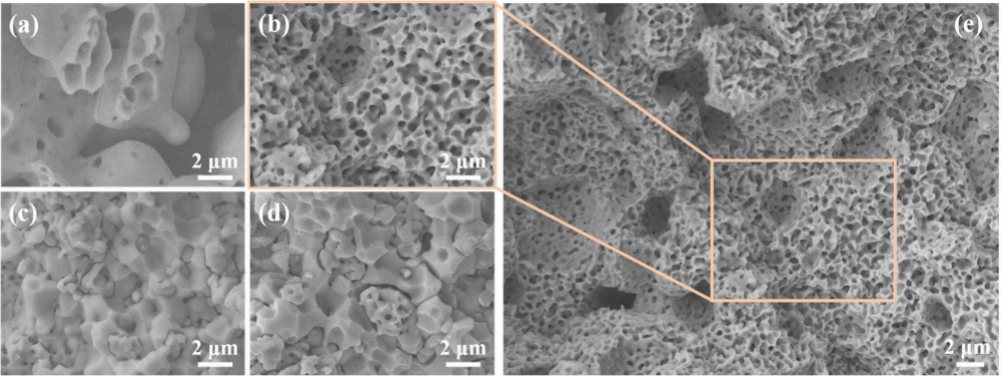
SEM images of the (a and b) Ni anode and (c and d) Ni-BZCYYb cermet anode after reduction under (a and c) 100% and (b and d) 8% H_2_ concentrations. (e) Zoomed-out view of the Ni anode reduced under 8% H_2_ (corresponds to panel b).

#### 3.1.3 Role of AFL

The metal-supported anode lacks the electronic–ionic conducting intertwined networks found in typical cermet anodes. Therefore, the incorporation of an AFL significantly enhances the performance of MS-PCFCs. AFLs with thicknesses of 3 and 10 μm were prepared, as shown in the SEM images (Figure S4). The microstructure of the AFL provides an extended reaction interface and abundant active sites at the gas–electrode–electrolyte junctions, thereby facilitating electrode reactions. The area-specific ohmic resistance (*R*_Ohm_) values for the cell with the 3 μm AFL were 0.19, 0.26, and 0.34 Ω cm^2^ at 700, 650, and 600 °C, respectively (Figure S5a). In contrast, increasing the AFL thickness to 10 μm (Figure S5b) resulted in lower *R*_Ohm_ values of 0.11, 0.13, and 0.17 Ω cm^2^ at 700, 650, and 600 °C, respectively. The *R*_Ohm_ values for the cell without an AFL were 0.20, 0.27, and 0.40 Ω cm^2^, which were significantly higher than that of the cell with a 10 μm AFL layer. The introduction of the AFL may suppress Ni diffusion from the metal substrate into the electrolyte, thereby mitigating the reduction in electrolyte conductivity. This speculation is supported by the EDS line-scan results in Figure S6, which reveal a slightly lower Ni content in the electrolyte with an AFL than in the cell without an AFL. The 10 μm AFL shows the lowest *R*_p_ of 0.13 Ω cm^2^ at 700 °C, while the 3 μm AFL exhibits a larger *R*_p_ of 0.5 Ω cm^2^, suggesting that insufficient thickness limits the continuity of the electrochemically active region and weakens interfacial kinetics (Figure S5c). The cell without an AFL presents a higher *R*_p_ of 0.2 Ω cm^2^, highlighting the importance of an adequately thick AFL in reducing fuel–electrode polarization. These results indicate that the porous AFL serves both to promote anode electrochemical activity and to moderate interfacial mismatch and Ni diffusion effects between the metal support and the electrolyte.

### 3.2 Factors Governing the Conductivity of the Electrolyte

#### 3.2.1 Ba Evaporation

Increased sintering temperatures lead to significant Ba evaporation, owing to the substantially lower enthalpy for Ba evaporation (142 kJ mol^–1^) in comparison to those of other cations (581.6 kJ mol^–1^ for Zr, 414 kJ mol^–1^ for Ce, and 363 kJ mol^–1^ for Y).^34^ This evaporation can lead to undesirable effects, including stoichiometric imbalance and the formation of secondary phases.^35,36^ Given the necessity for higher sintering temperatures during the fabrication of thin-film electrolytes for full cell assembly, various Ba species, Ba_*x*_Zr_0.1_Ce_0.7_Y_0.1_Yb_0.1_O_3−δ_ (*x* = 0.98, 1.00, 1.02, 1.04, and 1.06), were introduced into the electrolyte powder during synthesis. This addition was aimed at mitigating potential Ba deficiencies and achieving a stoichiometric composition of BZCYYb. The phase structures of the synthesized powders of Ba_*x*_Zr_0.1_Ce_0.7_Y_0.1_Yb_0.1_O_3−δ_ were characterized using X-ray diffraction. No discernible secondary phases were observed (Figure 2a). XRD patterns for excess Ba exhibited remarkable similarity, although the peak intensity was notably higher for the Ba-rich powder. Moreover, the BZCYYb (002) peak of 0.98Ba sample exhibited a discernible shift toward a larger angle (Figure 2b), indicative of a reduction in lattice parameters attributed to Ba depletion.^37^

**Figure 2:**
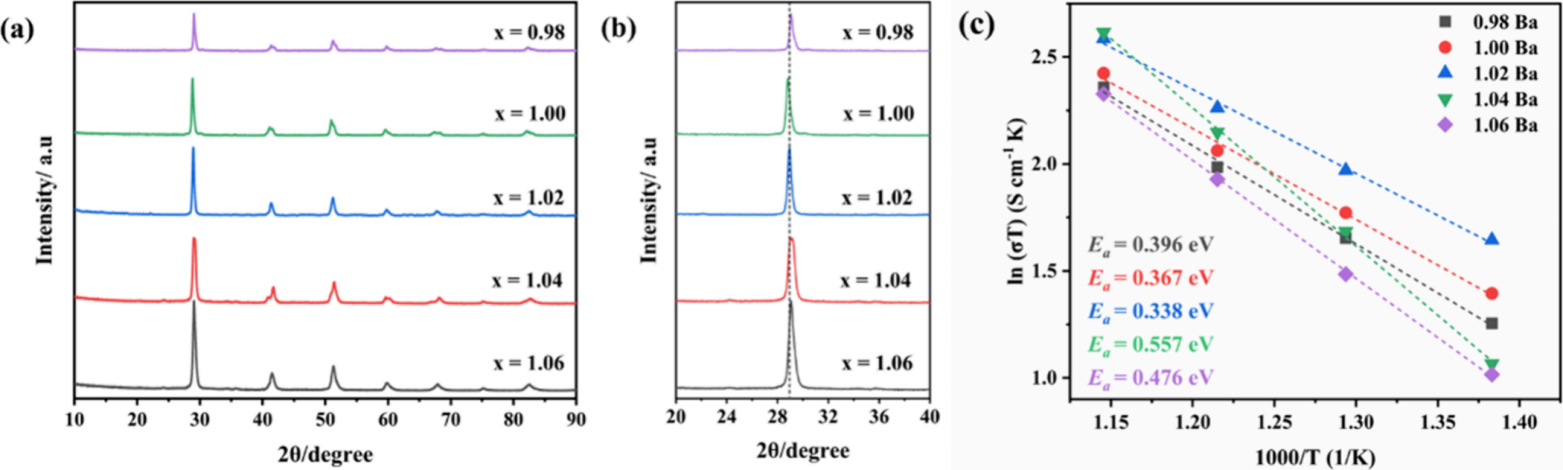
XRD patterns of Ba_*x*_Zr_0.1_Ce_0.7_Y_0.1_Yb_0.1_O_3−δ_ (*x* = 0.98, 1.00, 1.02, 1.04, and 1.06) powders sintered at 1150 °C for 10 h: (a) in the full range, (b) a magnified view, and (c) conductivity for the BZCYYb electrolyte with different Ba contents under a 40 vol % H_2_O humidified air atmosphere.

The influence of the varying Ba content on the electrolyte conductivity was measured by impedance spectra under 40 vol % H_2_O humidified air atmospheres at 450–600 °C. Three BZCYYb pellet samples with different Ba contents were sintered at 1375 °C for 10 h. Figure 2c presents the Arrhenius plots of proton conductivity for Ba_*x*_Zr_0.1_Ce_0.7_Y_0.1_Yb_0.1_O_3−δ_ electrolytes from 450 to 600 °C. The conductivity increases with Ba content from 0.98 to 1.02 but decreases with an increase in the Ba content to more than 1.02. Additionally, the activation energy (*E*_a_) of the excess 1.02 Ba sample is significantly lower than that of Ba_*x*_Zr_0.1_Ce_0.7_Y_0.1_Yb_0.1_O_3−δ_ (*x* = 0.98, 1.00, 1.04, and 1.06). This improvement can be attributed to the 2% excess Ba effectively compensating for Ba evaporation during high-temperature sintering, thereby maintaining the stoichiometry of the BZCYYb electrolyte. When the Ba content is increased beyond 1.02, Ba enrichment/volatilization at grain boundaries forms nanoscale/amorphous Ba-rich boundary layers and space-charge regions, which increase grain boundary resistance and hinder the continuity of proton conduction pathways, thereby impairing conductivity.^34,38–40^ Consequently, 1.02 Ba was applied to the MS-PCFC system to enhance the operational performance and stability of the cell.

#### 3.2.2 Ni Diffusion

Ni diffusion into perovskite-type electrolyte BZCYYb from the metal support will decrease the protonic conductivity.^41–43^ However, Ni can also function as a sintering aid for BZCYYb. To study the effect of Ni diffusion on BZCYYb during sintering, one green BZCYYb pellet was sandwiched between two sintered NiO disks that act as the source of nickel diffusion during the sintering process. The conductivity of electrolyte samples sintered at 1350 °C was calculated based on the impedance spectra measured under air with 40% steam. As shown in Figure 3a, the sample without Ni diffusion exhibited a higher conductivity, reaching 8 × 10^–3^, 10 × 10^–3^, 15 × 10^–3^, and 20 × 10^–3^ S cm^–1^ at 450, 500, 550, and 600 °C, respectively. SEM images of the cross section of the electrolyte pellets, sintered with and without Ni diffusion, are presented in Figure 3b,c. The sample with Ni diffusion displayed a denser bulk structure and larger crystal grains compared to the samples without. It also exhibited a higher activation energy of 0.445 eV, versus a value of 0.420 eV without Ni diffusion. It has been reported that a grain boundary consists of one grain boundary core and two adjacent space-charge layers and the grain boundary core displays a positive potential due to the enriched oxygen vacancy.^44^ The protons in space-charge layers are diluted due to electrostatic interaction, theoretically leading to a decrease in proton concentration in grain boundaries, but the grain size and grain boundary might not be the only cause of the observed trends. The reduction in proton concentration is likely another reason for the decreased conductivity of the electrolyte with Ni diffusion.^45^ It has been shown that sintering aids can reduce proton uptake, thereby decreasing the material’s hydration ability.^46^

**Figure 3:**
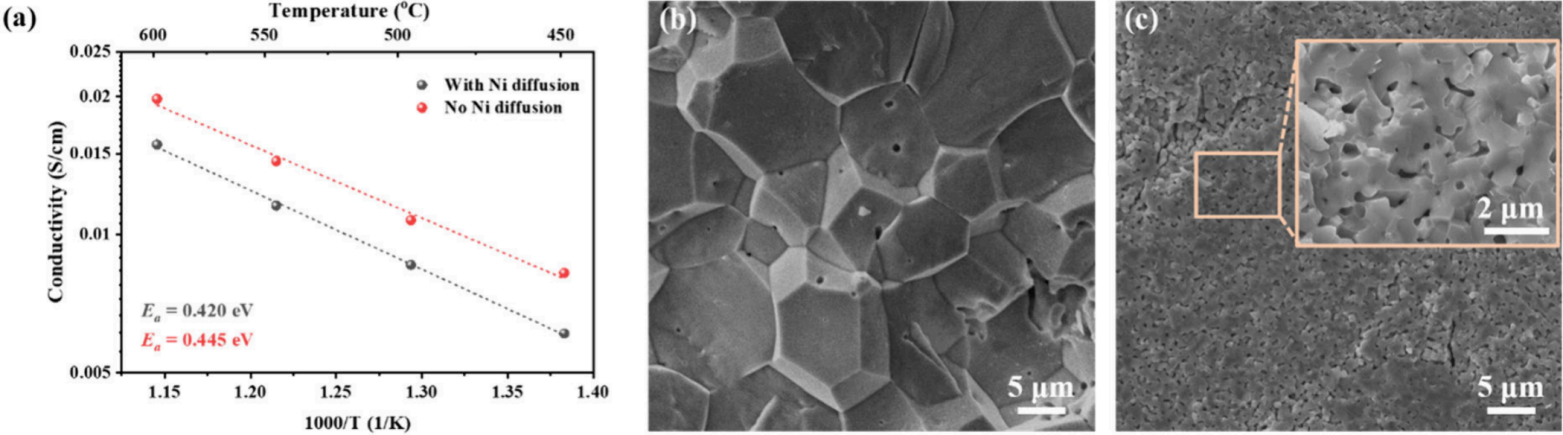
(a) Conductivity for the bulk electrolyte of BZCYYb sintered with and without Ni diffusion, measured in air with 40% steam. SEM images of the cross section of electrolytes pellet samples sintered at 1350 °C for 5 h (b) with and (c) without Ni diffusion after measurement.

On the basis of the above results from stand-alone electrolyte pellets, it is evident that Ni diffusion significantly impacts the electrochemical performance of the electrolyte, even when NiO is not deliberately added to the electrolyte. To further confirm the influence of Ni diffusion on the full cell electrolyte during cosintering, TEM imaging and elemental mapping of the cross section of Ni-supported single cells after testing were conducted, as shown in Figure 4. The results demonstrate that Ni migration from the support into the BZCYYb electrolyte occurred during cosintering. This migration is accompanied by a deficiency of Ba at the grain boundaries, along with segregation of Y and Yb from the proton-conducting phase. Such changes are detrimental to electrolyte performance, given that the conductivity of BZCYYb is highly sensitive to these contents. In this regard, decreasing the sintering temperature of the electrolyte would limit the diffusion of Ni from the support and reduce Ba evaporation.

**Figure 4:**
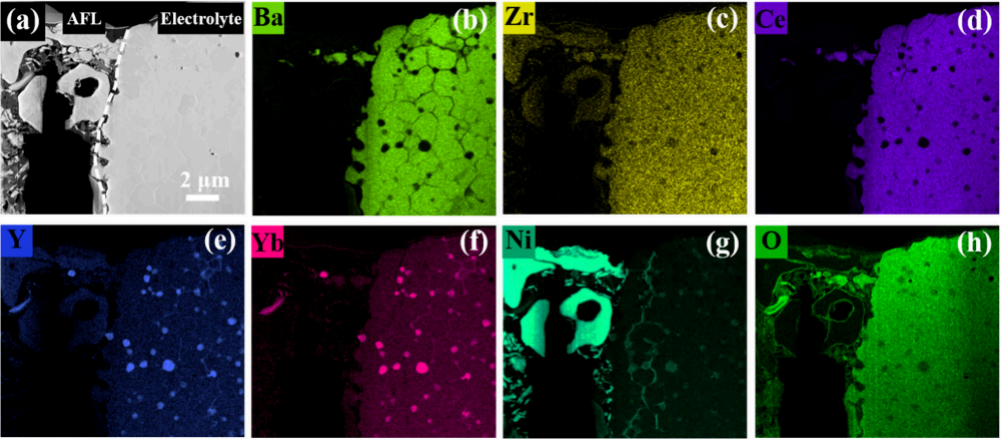
(a) TEM images of the Ni||Ni-BZCYYb||BZCYYb||PNO cell. (b–h) Element mapping of Ba, Ce, Zr, Y, Yb, Ni, and O, respectively, after operation and element mapping.

#### 3.2.3 Sintering Temperature

Different sintering temperatures on the electrolyte conductivity in the Ni-supported single cell were investigated. An Arrhenius plot (Figure 5a) for the conductivity of the thin-film electrolyte was generated from impedance measurements (Figure S7). The electrolyte sintered at 1250 °C exhibited notably lower total conductivity compared to those sintered at 1320, 1350, and 1380 °C. This difference can be attributed to the presence of closed pores and considerably smaller crystal grains (0.4–2 μm) at 1250 °C, which lead to decreased ionic conductivity^47^ (Figure 5b). At 1380 °C, as shown in Figure 5e, the higher sintering temperature promoted the growth of larger crystal grains (2–4 μm) but resulted in a higher activation energy value (0.422 eV) compared to that at 1350 °C (0.352 eV). This was attributed to accelerated Ni diffusion into the BZCYYb electrolyte layer at the high sintering temperature of 1380 °C, which degraded protonic conductivity. Notably, the electrolyte sintered at 1320 °C exhibited the highest conductivity within the temperature range of 600–700 °C, albeit with a larger activation energy (*E*_a_ = 0.495 eV).

**Figure 5:**
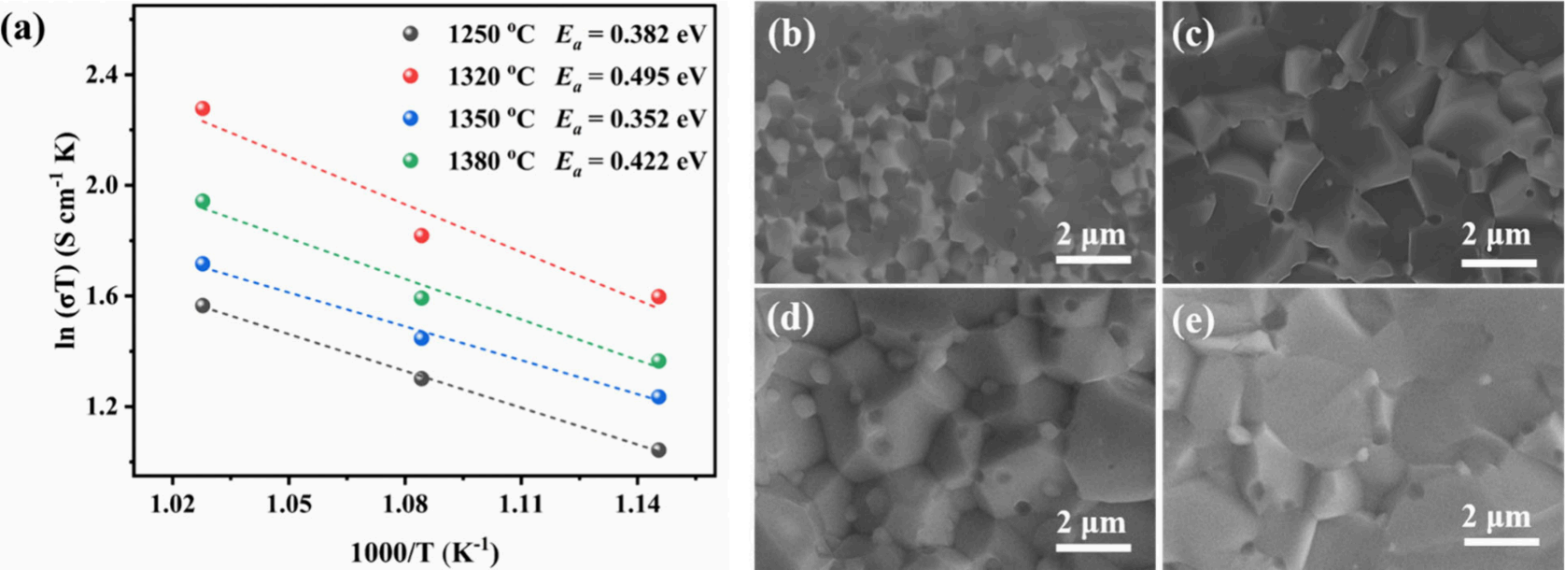
(a) Arrhenius plots of BZCYYb in Ni||Ni-BZCYYb||BZCYYb||PNO single cells with the anode substrate and electrolyte sintered at 1250, 1320, 1350, and 1380 °C measured under dry H_2_ at 600, 650, and 700 °C. SEM images of the cross section of the electrolyte’s samples cosintered with an anode substrate at (b) 1250, (c) 1320, (d) 1350, and (e) 1380 °C after measurement.

### 3.3 Full Cell Performance

The electrochemical performance of the Ni||Ni-BZCYYb||BZCYYb||PNO single cells fabricated using optimal parameters discovered above was evaluated at different operating temperatures (Figure 6). As shown in Figure 6a, *R*_Ohm_ values of 0.17, 0.13, and 0.11 Ω cm^2^ were found at 600, 650, and 700 °C, respectively. Concurrently, the total polarization resistance (*R*_p_) values were 0.31, 0.20, and 0.14 Ω cm^2^, respectively. The peak power densities of the cell were 0.46, 0.72, and 1.05 W cm^–2^ at 600, 650, and 700 °C, respectively (Figure 6b). This performance far exceeds previously reported results for PCFCs using the Ni–Fe metal-supported anode at intermediate temperatures (773–973 K).^48^ Subsequently, a durability examination of the full cell was conducted, and the results are presented in Figure 6c. The constant current density was determined from the H_2_ *I–V* curve as the current corresponding to an initial cell voltage of 0.7 V at 600 °C, ensuring a well-defined and reproducible operating condition. Under these conditions, the cell was continuously operated at 600 °C, sustaining a constant current of 0.55 A cm^–2^ for more than 650 h. While there was minor fluctuation in power output, no discernible degradation was noted throughout the duration of operation, suggesting the electrochemical performance of the cell remained stable.

**Figure 6:**
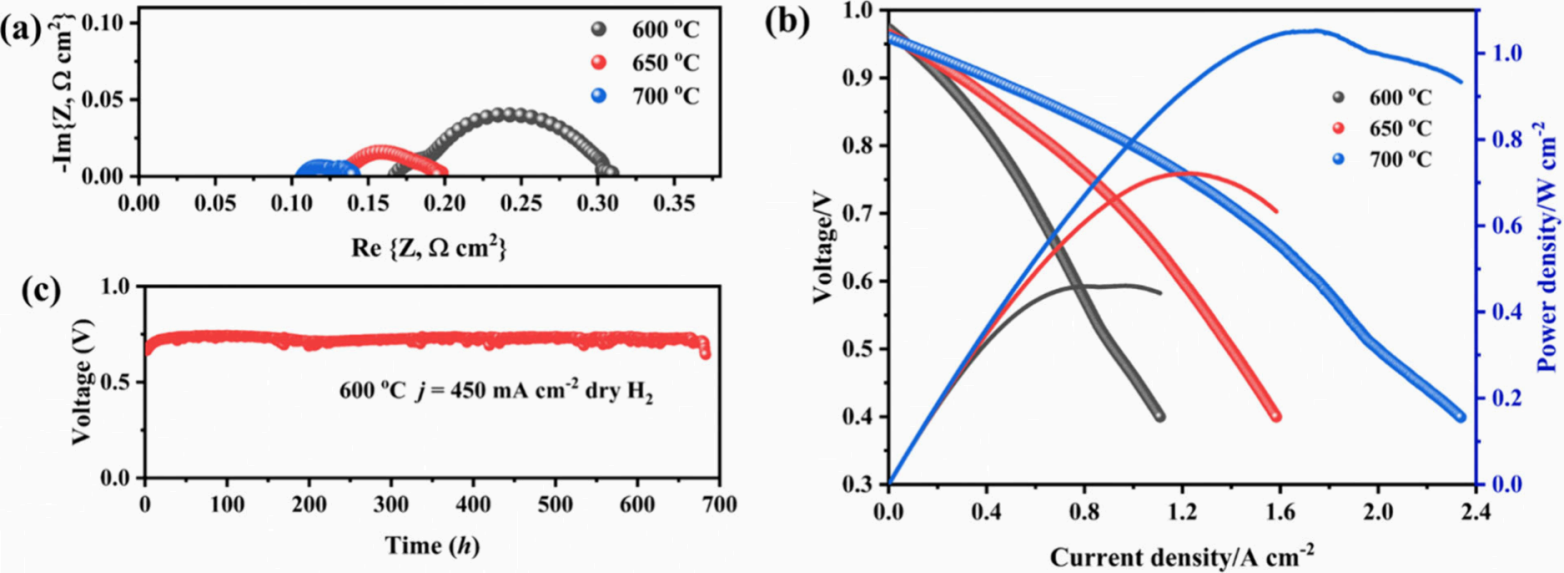
(a) EIS, (b) *I–V* and power density curves, and (c) durability testing of the Ni||Ni-BZCYYb||BZCYYb||PNO full cell operated at 600 °C under a constant current of 0.45 A cm^–2^ in H_2_ as fuel.

When NH_3_ is used as fuel, a discernible increase in both *R*_Ohm_ and *R*_p_ is observed (Figure S8), accompanied by the emergence of structural cracks within the cell. Previous studies have substantiated that the exposure to ammonia leads to the coarsening and agglomeration of nickel, contributing to the accelerated deterioration of anodic performance over time.^26,49,50^ To address this challenge, we introduced an easily recyclable upstream NH_3_ predecomposition design within the anode architecture. A small amount of nickel catalyst powder was placed in the fuel gas conduit near the anode. To confirm the effectiveness of the NH_3_ predecomposition design, the gas passing though the fuel conduit was collected at 600 °C and analyzed by gas chromatography (GC). It shows that the NH_3_ conversion rate is 86%, demonstrating the feasibility and high efficiency of this predecomposition device. During operation, 80 sccm of NH_3_ was introduced to the anode side. The performance characteristics of the cell are shown in Figure 7. At an operating temperature of 600 °C, *R*_Ohm_ and *R*_p_ exhibited values of 0.296 and 0.652 Ω cm^2^, respectively, as illustrated in Figure 7a. The resulting peak power density for the cell was 0.48 W cm^–2^ at 650 °C (Figure 7b). Compared to the aforementioned performance using H_2_ as fuel, a slight decrease is observed. For the same cell, the electrochemical impedance spectra and current–voltage curves for cells operated under NH_3_ fuels yielded results comparable to those with H_2_ (Figure S9).

**Figure 7:**
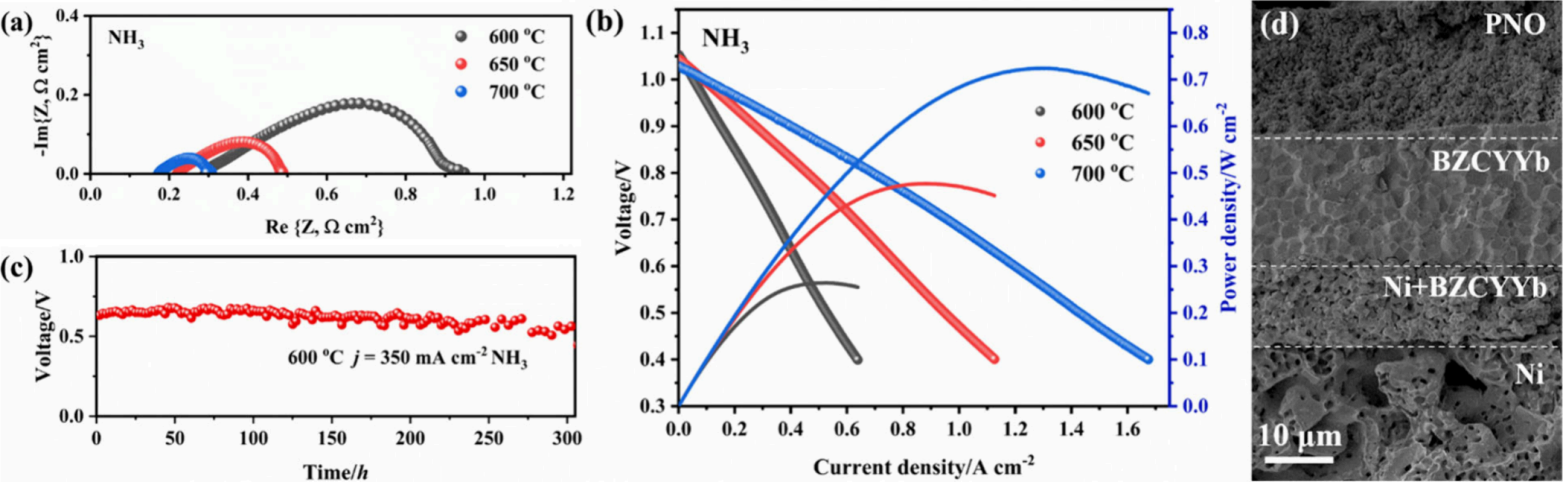
(a) EIS, (b) *I–V* and power density curves, and (c) long-term durability testing at 600 °C under a constant current of 0.35 A cm^–2^ of the Ni||Ni-BZCYYb||BZCYYb||PNO full cell under NH_3_ as fuel. (d) SEM images of the cross section of the single cell after operation.

In the present MS-PCFC configuration, upstream introduction of a Ni-packed bed significantly reduced the in situ decomposition load on the electrode. NH_3_ utilization involves sequential gas diffusion, catalytic decomposition into N_2_ and H_2_, and rapid H_2_ dissociation to yield protons and electrons.^26,51,52^ To decouple and assign the electrochemical and mass-transport contributions under both H_2_ and NH_3_ fuels, distribution of relaxation time (DRT) analysis was performed on the impedance data at 600–700 °C (Figure S10). As shown in Figure S10a, the spectra exhibit two main features under H_2_ fuel with a dominance midfrequency (MF) peak (∼10^2^ Hz) associated with charge-transfer kinetics at the fuel electrode, and a high-frequency (HF) peak (∼10^3^–10^4^ Hz) linked to interfacial proton transfer. For NH_3_ operation (Figure S10b), additional low-frequency features (<10 Hz) emerge under NH_3_ fuel, particularly at lower temperatures, which was attributed to residual ammonia decomposition and N_2_–H_2_ gas phase diffusion within the porous electrode. A direct comparison at each temperature (Figure S10c-e) shows that NH_3_-fed operation exhibits a slightly larger contribution at 600 °C, which can be attributed to residual decomposition and gas diffusion, but this difference decreases markedly at 650 and 700 °C. Overall, the DRT results showed that the electrochemical behavior of H_2_ and NH_3_ was largely comparable, which confirmed that the upstream Ni-packed decomposition unit effectively mitigates additional conversion and transport limitations, allowing NH_3_-fed operation to approach the intrinsic charge-transfer performance of H_2_.

The durability assessment using NH_3_ is depicted in Figure 7c. In accordance with the constant-current selection criterion established in the H_2_ durability test, the cell was operated at 600 °C for 300 h at a fixed current density of 0.35 A cm^–2^. The microstructural features of the full cell after operation are captured in the SEM images in Figure 7d and Figure S11, where the individual layers of the Ni||Ni-BZCYYb||BZCYYb||PNO multilayer structure can be clearly distinguished with thicknesses of approximately 600, 10, 18, and 17 μm. Notably, robust bonds are discernible at the cathode–electrolyte, electrolyte–anode function layer, and anode function layer–anode interfaces, confirming the structural integrity of the cell. The cell exhibited negligible performance decay within the first 100 h of operation. Between 100 and 200 h, the degradation rate was approximately 2.7%, and it further increased to about 9.8% during the period from 200 to 300 h. To investigate the origin of the degradation during long-term operation, we compared the EIS spectra before and after long-term operation. As shown in Figure S12, the EIS data after long-term operation were fitted using the equivalent circuit model. The results show that the total polarization resistance remains approximately 0.6 Ω cm^2^, indicating no significant change in cell polarization during long-term operation. In contrast, the ohmic resistance increased noticeably from 0.3 to 0.4 Ω cm^2^, suggesting that the performance degradation mainly originated from the increased ohmic resistance. The DRT analysis is consistent with this conclusion, as it resolves the corresponding frequency-domain contributions without indicating a substantial increase in the overall polarization processes. To further elucidate the structural stability of the Ni anode under different operating atmospheres, XRD characterization was performed after long-term operation. The XRD patterns of the Ni anode after long-term operation under both NH_3_ and H_2_ atmospheres are shown in Figure S13. All diffraction peaks can be indexed to metallic Ni (PDF#04-0850), and no additional reflections corresponding to nickel nitrides or secondary phases were observed.^53^ This clearly demonstrated that even after prolonged exposure to ammonia, the Ni anode preserved its metallic structure, benefiting from the upstream predecomposition design that suppresses nitride formation.

To assess possible Ni migration during operation with ammonia fuel, cross-sectional SEM imaging combined with EDS line scanning was performed on both a fresh cell and a tested cell (Figure S14). In the fresh sample, the Ni signal is strongly localized within the metal support and decreases across the anode functional layer, while remaining near the baseline level in the electrolyte region. After long-term measurement under ammonia-containing fuel, the Ni remains confined to the support–AFL region and no measurable Ni enrichment is observed on the electrolyte side. The results confirm that Ni diffusion into the electrolyte is negligible under the present operating conditions and does not contribute significantly to the observed performance changes.

The redox behavior of the metal-supported cell is shown in Figure S15. After one redox cycle, the Nyquist plot at 700 °C reveals an increase in ohmic resistance from 0.33 to 0.55 Ω cm^2^, whereas the polarization resistance remains unchanged at 0.18 Ω cm^2^, suggesting that the redox excursion primarily influences the conduction pathways in the metal-supported structure while leaving the electrode reaction kinetics largely intact. The slight OCV decrease after redox cycling is attributed to microstructural changes caused by Ni oxidation and subsequent rereduction. The associated volume expansion and contraction may induce minor interfacial defects or microcracks.^54^ Consistently, the increased ohmic resistance after redox cycling suggests possible electrolyte or interfacial damage, which may promote gas leakage and reduce the effective oxygen partial pressure gradient.^55,56^ Consistent with the increase in ohmic resistance, the *I–V* and power density curves show a reduced peak power density and a slight decrease in OCV. Although performance degradation is observed, the cell remained operational after one redox cycle without catastrophic structural or interfacial failure, indicating that the porous Ni metal support and graded anode configuration can survive a single redox event under the applied conditions.

Given the limited availability of directly comparable data for purely metal-supported PCFCs operated under identical electrolytes and conditions, Table S3 places the present results within the broader context of NH_3_-fed PCFC studies. The Ni-supported anode cell developed in this work demonstrates stable operation over the 300 h test period without catastrophic degradation. The optimized cosintering of the metal support and electrolyte offers good mechanical compatibility, contributing to the preservation of interfacial integrity during operation. In addition, the upstream ammonia predecomposition approach helps moderate the extent of Ni nitridation during long-term NH_3_ exposure, supporting stable cell behavior under the applied conditions.

## 4 Conclusion

In conclusion, this study investigated the fabrication and performance optimization of MS-PCFCs, with a focus on the anode substrate, electrolyte, and sintering conditions. The use of a Ni-supported anode material in MS-PCFCs was explored, and the intricate interplay among Ni diffusion, sintering temperature, and electrolyte performance was systematically examined.

The study demonstrated that the appropriate thickness of the AFL, optimized anodic reduction process, and Ni diffusion from the anode to the electrolyte during cosintering significantly influenced the electrochemical behavior of the single cell. As a result, the MS-PCFC achieved a high peak power density of 480 mW cm^–2^ at 650 °C when operated on NH_3_ fuel, outperforming various other NH_3_-powered fuel cells. Notably, the cells with this anode configuration operated at a current density of 0.35 A cm^–2^ at 600 °C for 300 h, demonstrating good stability for using ammonia as fuel.

In summary, this research contributes valuable insights into the intricate fabrication processes and performance optimization of MS-PCFCs, shedding light on the critical factors influencing their efficiency and stability. The findings provide a foundation for further advancements in metal-supported protonic ceramic fuel cell technology, offering potential applications in cleaner and more sustainable energy conversion systems.

## Supplementary Material



## Notes

Schematic diagram of the MS half cell, SEM characterization, OCV curves, *I–V* curve, EIS characterization corresponding to each electrochemical analysis, DRT analyses, XRD patterns, shrinkage comparison table, and performance comparison table (PDF)
