# Supplementary material for: High-Performance Metal-Supported Protonic Ceramic Fuel Cell Utilizing Ammonia-Derived Fuel
Source: ACS Appl Mater Interfaces. 2026 May 19;18(21):29810–9. doi: 10.1021/acsami.5c18211 (PMC13244364; doi:10.1021/acsami.5c18211)
Supplement: Supplementary file 1 [file am5c18211_si_001.pdf]

Supporting Information:

High-Performance Metal-Supported Protonic Ceramic Fuel Cell Utilizing Ammonia-Derived Fuel

Xuemei Li[1], Hanchen Tian[2], Bo Guan[3], Qingyuan Li[2], Lingfeng Zhou[1], Awa Kalu[1], Shaoshuai Chen[2], Xinyuan Zhu[2], Yu Xie[2], Siyuan Liu[1], Xingbo Liu[2]*, Wenyuan Li[1]*

[1]Chemical and Biomedical Engineering Department, Benjamin M. Statler College of Engineering Mineral Resources, West Virginia University, Morgantown, WV 26506, USA

[2]Mechanical Aerospace Engineering Department, Benjamin M. Statler College of Engineering Mineral Resources, West Virginia University, Morgantown, WV 26506, USA

[3]DOE National Energy Technology Laboratory, 3610 Collins Ferry Road, Morgantown, West Virginia, USA

*Corresponding Author, E-mail: xingbo.liu@mail.wvu.edu, wenyuan.li@mail.wvu.edu

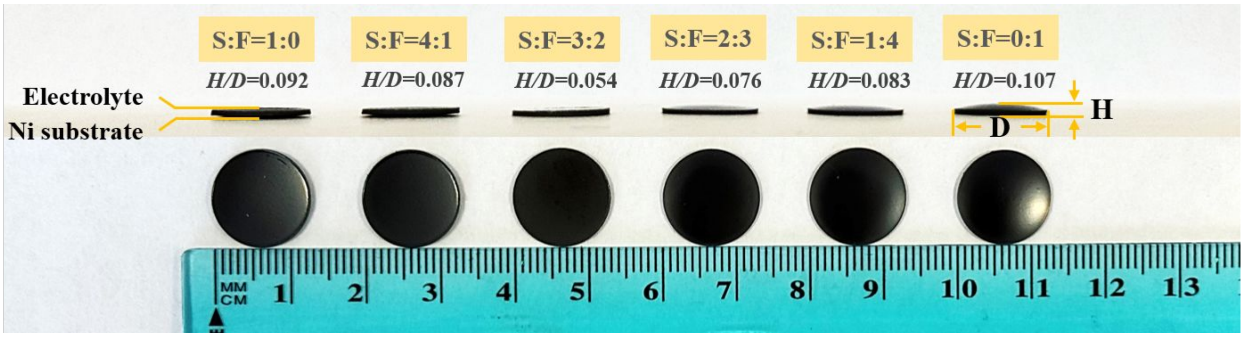

Fig. S1 The half-cell samples fabricated using NiO-S (coarse) and NiO-F (fine) powders with different mixing ratios (S:F = 1:0, 4:1, 3:2, 2:3, 1:4, and 0:1) after sintering under 1350 ^{o}C for 5.5h

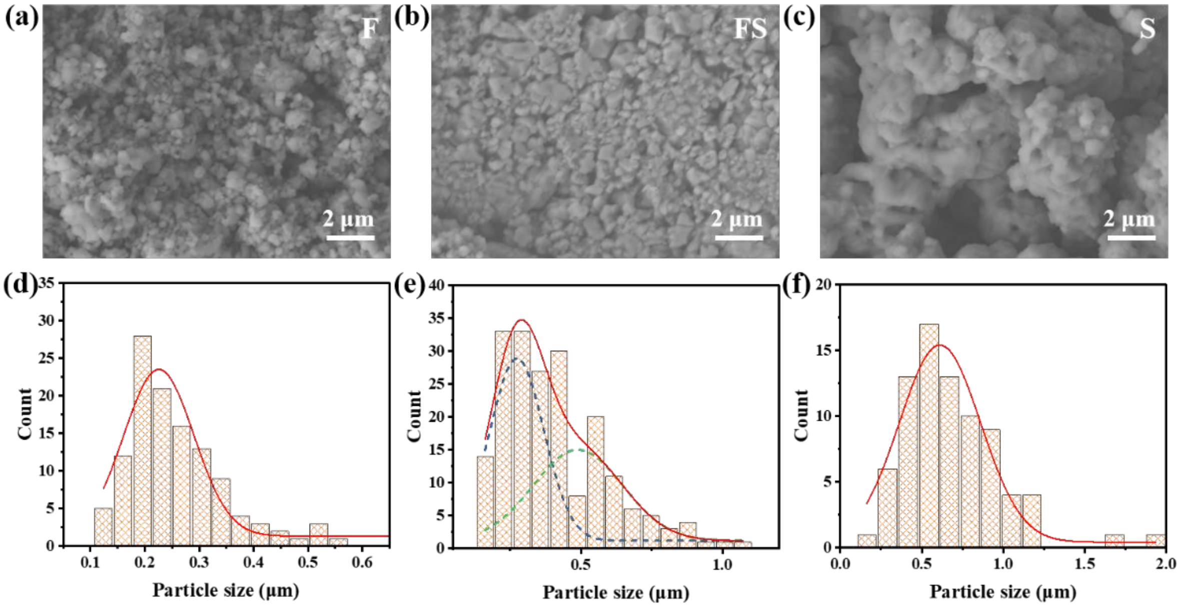

Fig. S2 SEM images of (a) fine NiO powder (F), (b) mixed NiO powder (FS, S:F = 3:2), and (c) coarse NiO powder (S), along with the corresponding particle-size distributions shown in (d-f).

(a)

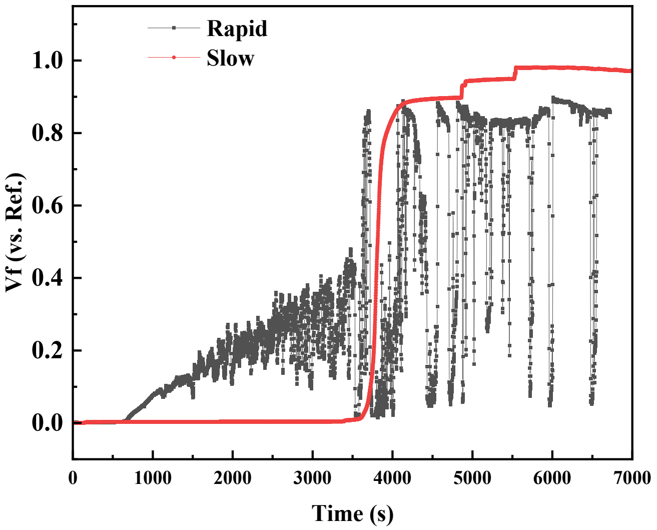

(b)

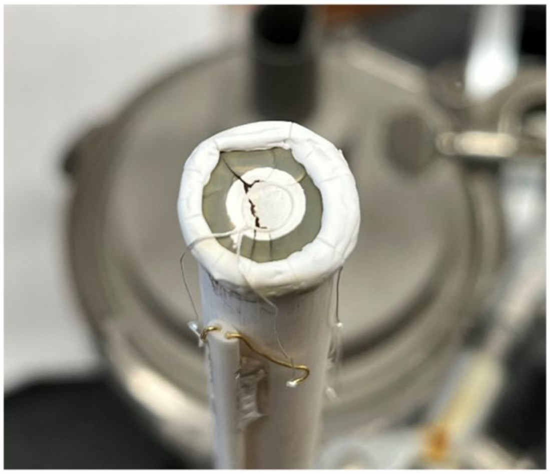

Fig. S3 (a) OCVs of the pure Ni-supported fuel cell samples with rapid and slow reduction process of anode and (b) picture of full cell after rapid reducing process.

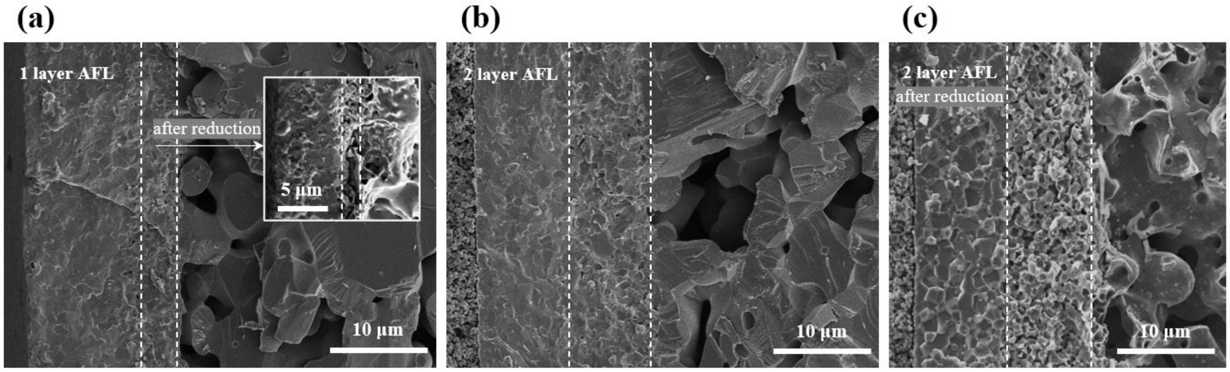

Fig. S4 SEM images of the cross-section of the cells spin-coated AFL at (a) 3 μm and (b and c) 10 μm. (c) is (b) after reduction and operation

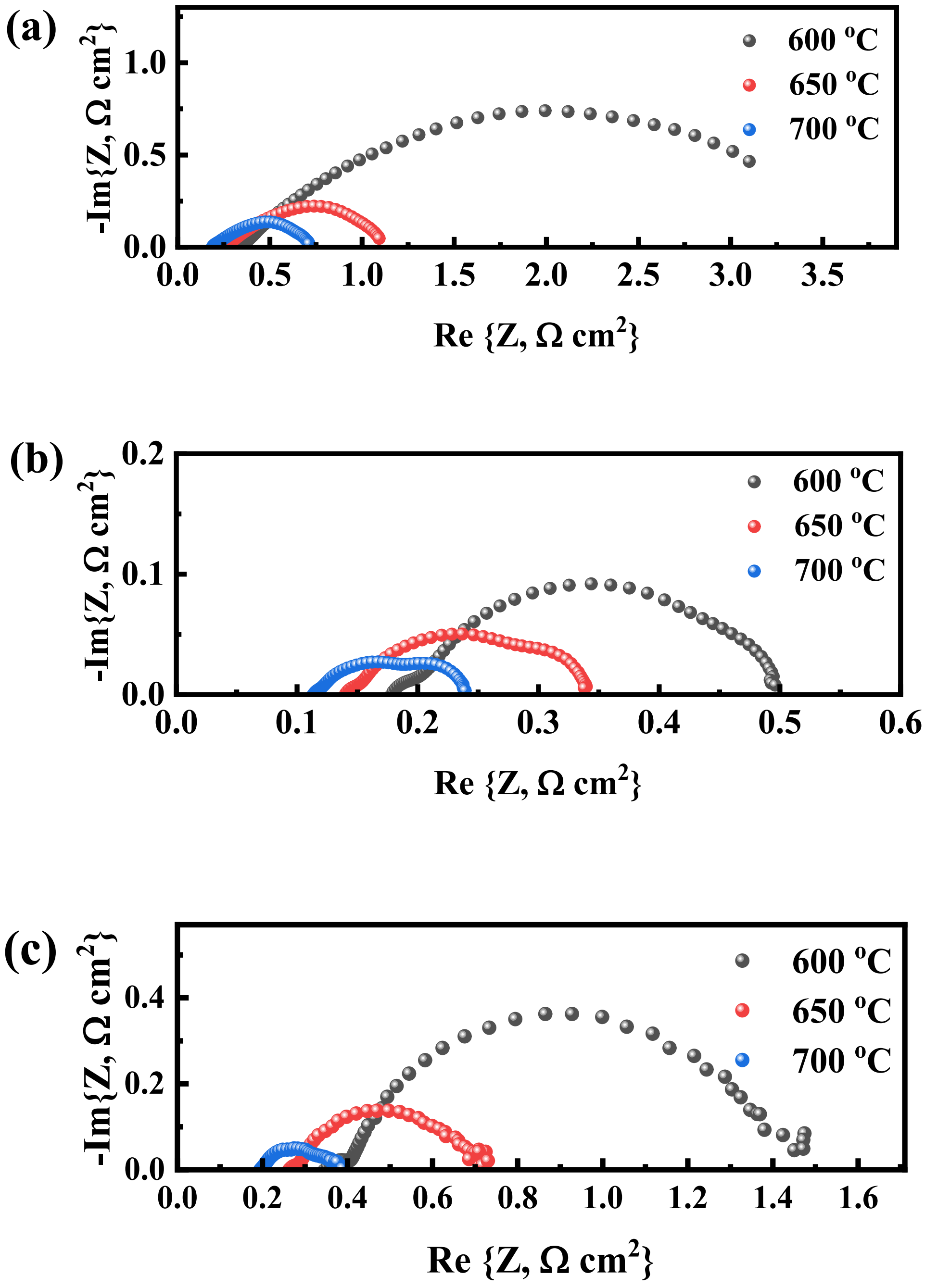

Fig. S5 EIS of Ni||Ni-BZCYYb||BZCYYb/PNO full cell with (a) 3 μm and (b) 10 μm thickness of the AFL, and (c) without AFL layer

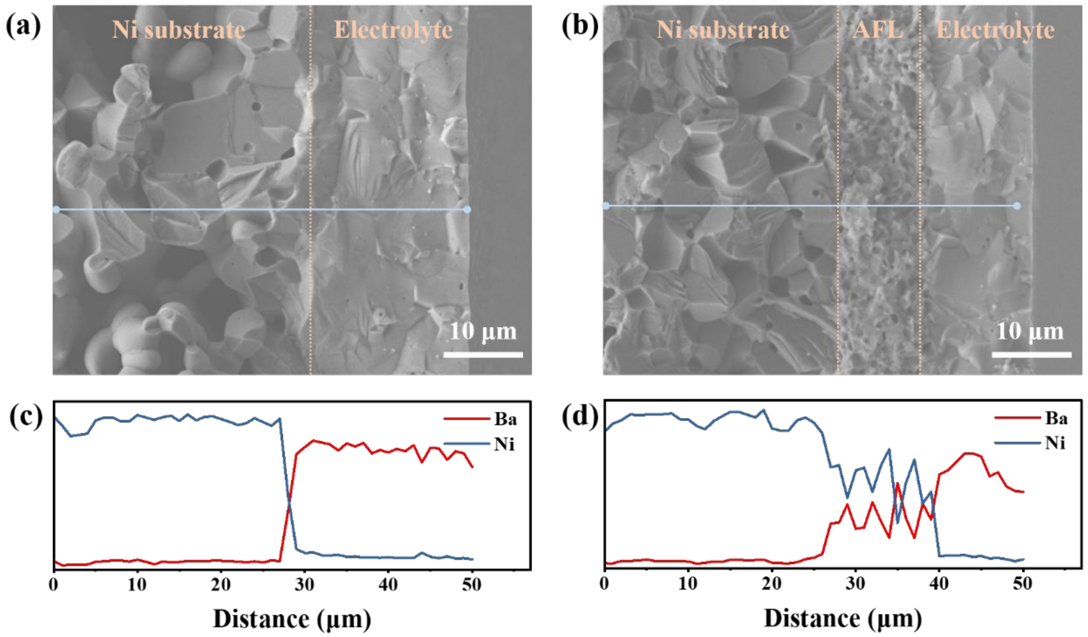

Fig. S6 Cross-sectional SEM images of the Ni substrate/electrolyte interface for cells (a) without an AFL and (b) with an AFL, with corresponding EDS line-scan profiles shown in (c) and (d).

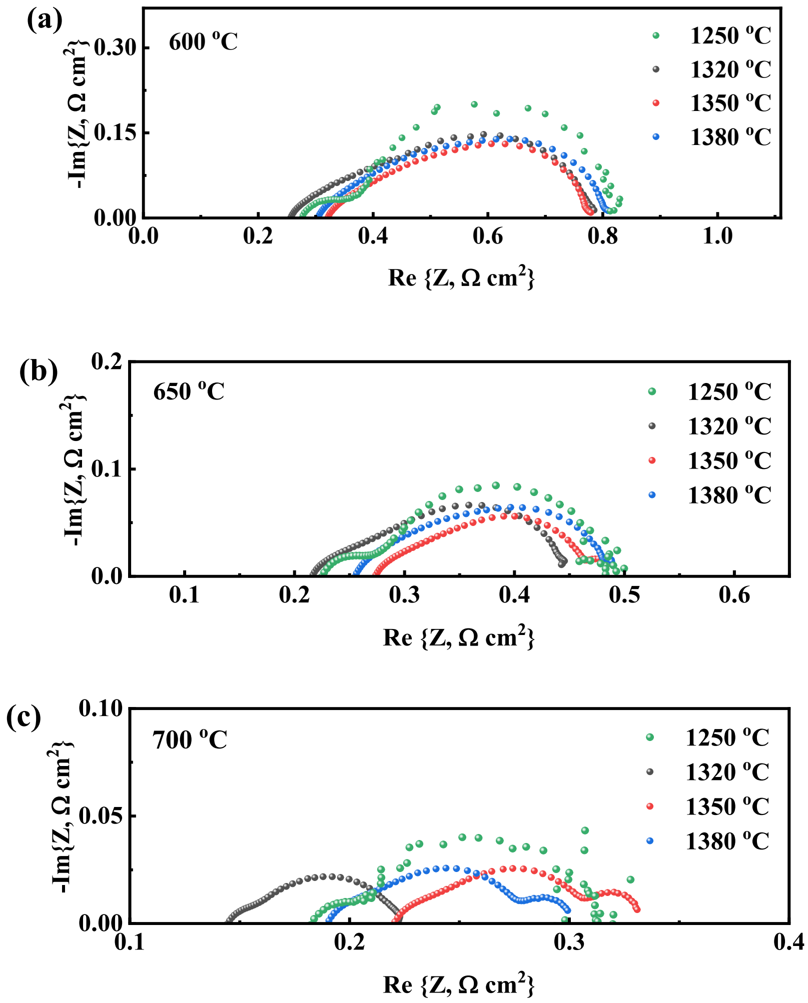

Fig. S7 EIS plots of Ni||Ni-BZCYYb||BZCYYb||PNO full cells with the anode substrate and electrolyte co-sintered at 1250, 1320, 1350 and 1380 ^{o}C measured at (a) 600, (b) 650 and (c) 700 ^{o}C

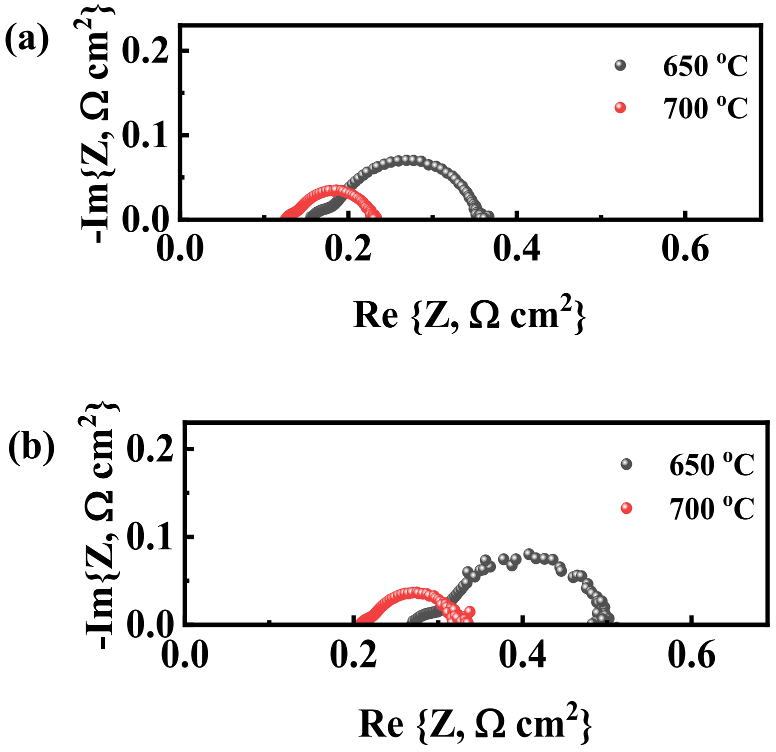

Fig. S8 (a) EIS of Ni||Ni-BZCYYb||BZCYYb||PNO full cell under (a) dry H_2 and (b) NH_3 as fuels

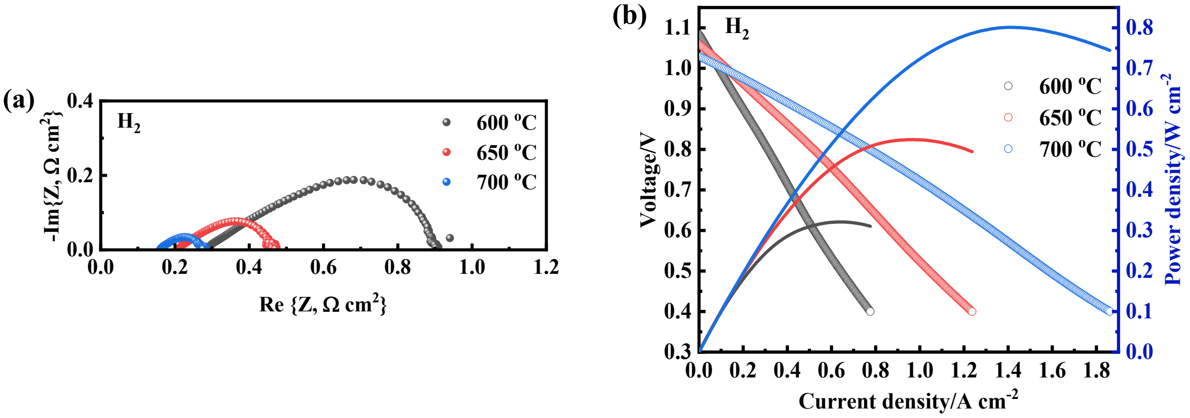

Fig. S9 (a) EIS and (b) *I-V* and power density curves of Ni||Ni-BZCYYb||BZCYYb||PNO full cell under H_2 as fuels

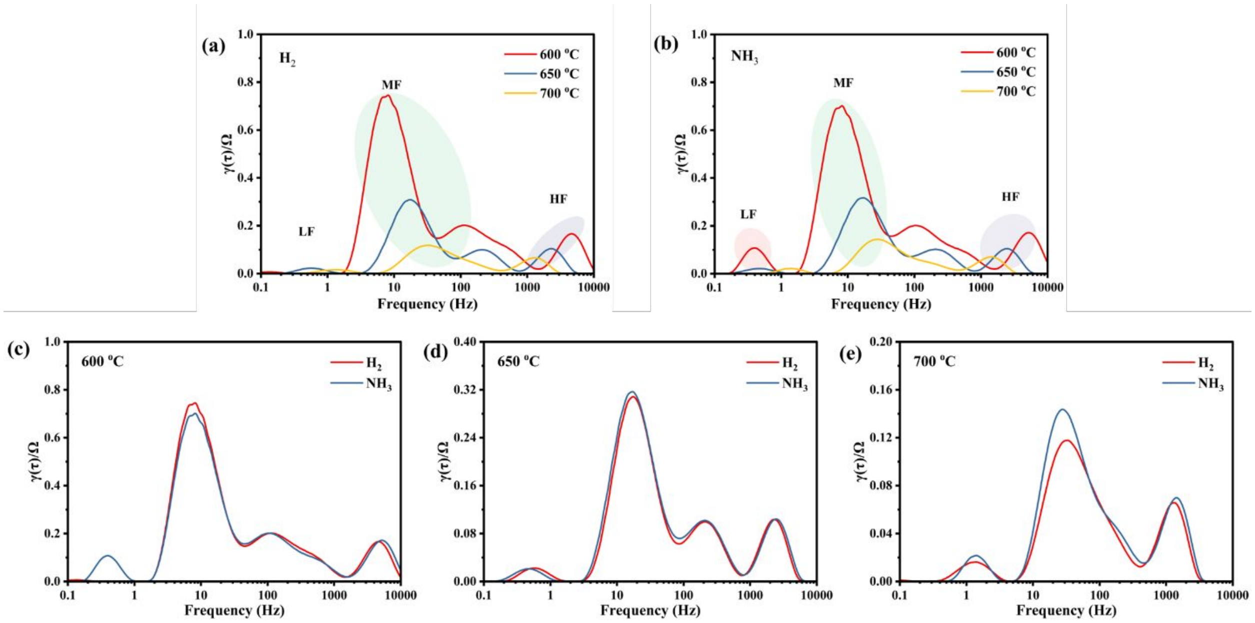

Fig. S10 DRT analyses of MS-PCFC under (a) H_2 and (b) NH_3 in fuel electrode side at 600-700 °C, and direct H_2-NH_3 comparisons at (c) 600 °C, (d) 650 °C, and (e) 700 °C

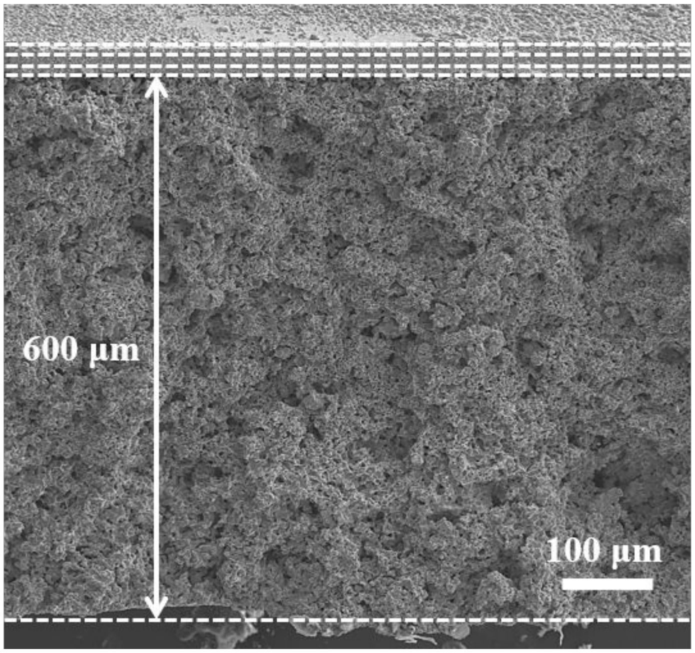

Fig. S11 Cross-sectional SEM image of the Ni|Ni–BZCYYb|BZCYYb|PNO multi-layer structure after operation. Dashed lines indicate the interfaces between adjacent layers

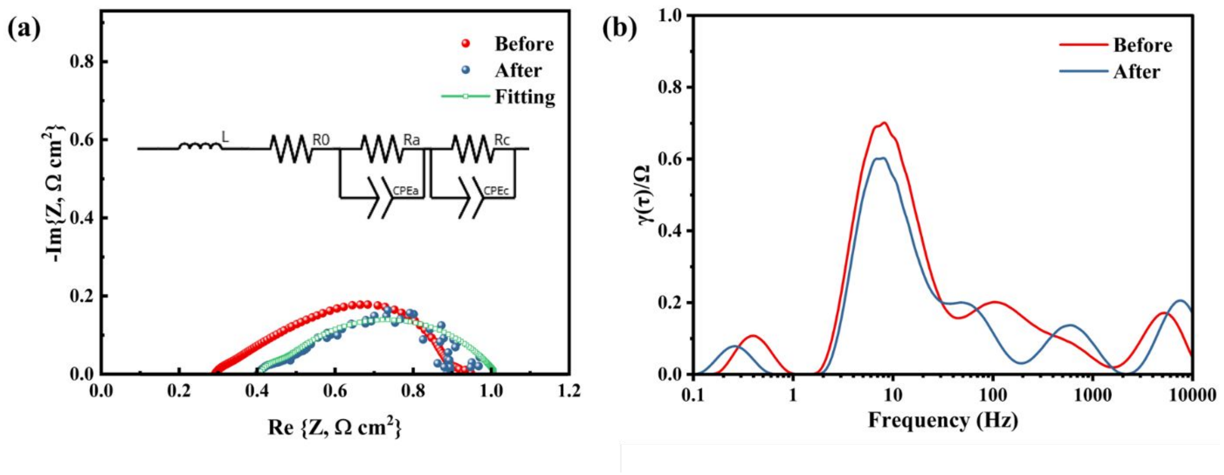

Fig. S12 (a) EIS and two-arc profile fitting, and (b) DRT spectra of the full cell before and after long-term operation under NH_3 atmosphere

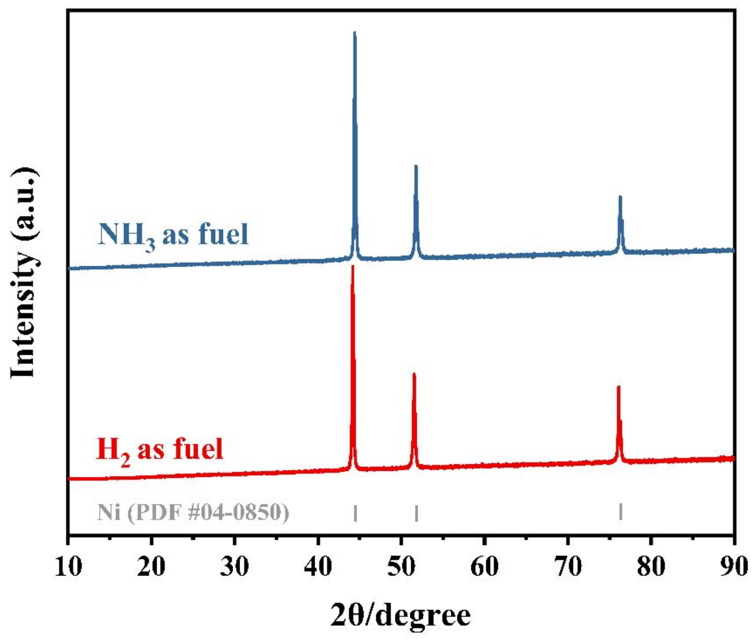

Fig. S13 XRD patterns of the Ni anode after long-term operation under H_2 and NH_3 atmospheres

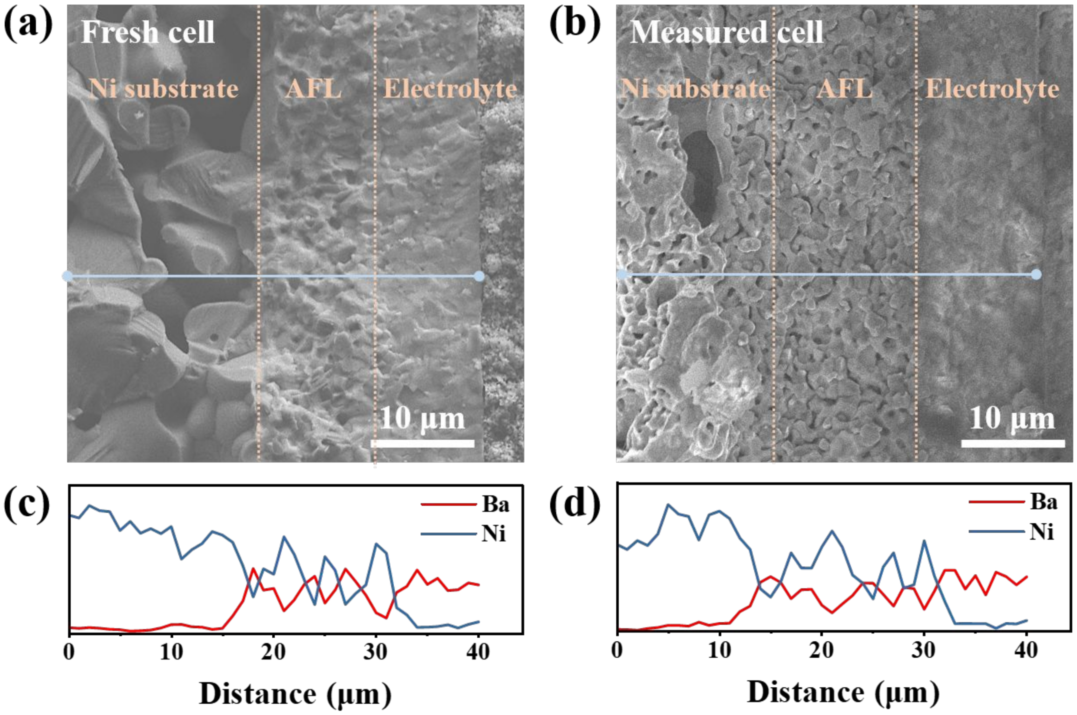

Fig. S14 (a and b) Cross-sectional SEM images and (c and d) corresponding EDS line scans of Ni and Ba for (a and c) the fresh cell and (b and d) the cell after ammonia-fuel measurement

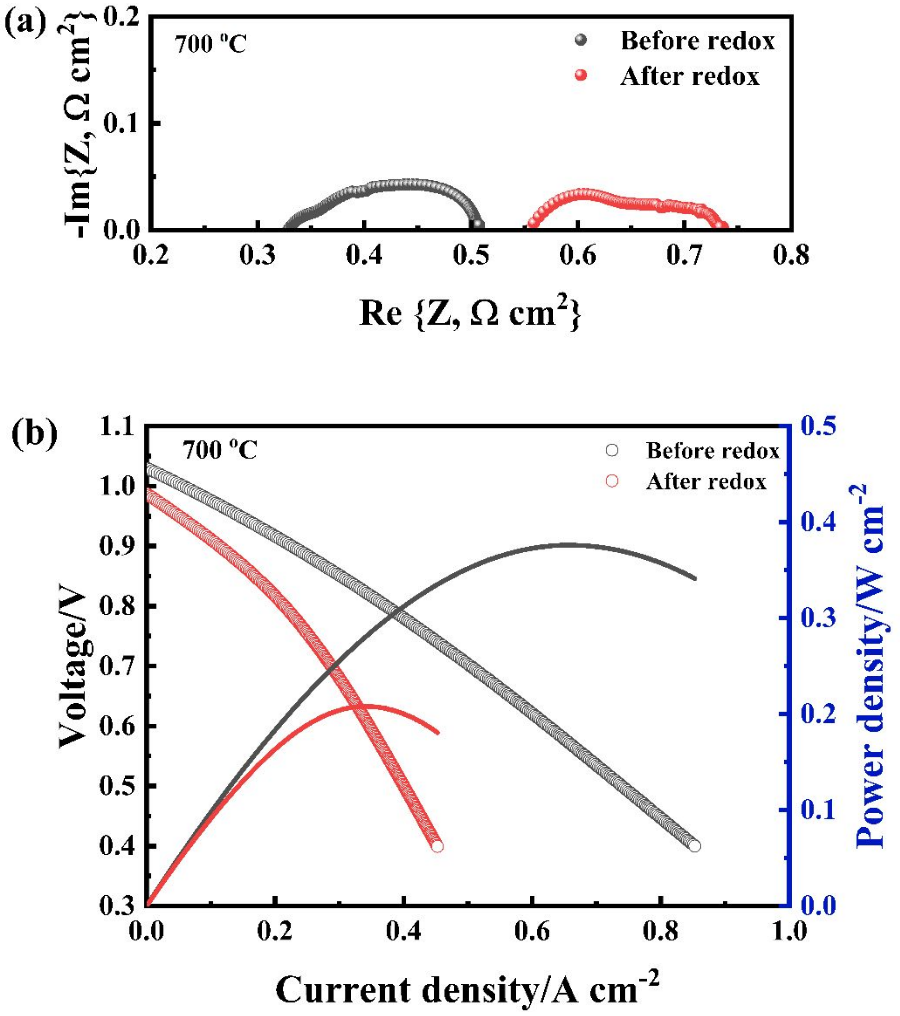

Fig. S15 Electrochemical impedance spectra, I-V curve and current-power density curve of the metal-supported cell at 700 °C before and after a redox cycle under dry H_2 in fuel electrode side and dry air in air electrode side

Table S1 Measured diameter, maximum deflection height, and calculated H/D ratio of samples fabricated using standard coarse NiO (S) and fine NiO (F) powders with different mixing ratios

	S:F=1:0	S:F=4:1	S:F=3:2	S:F=2:3	S:F=1:4	S:F=0:1
Diameter (mm)	13.16	13.16	12.95	13.11	13.11	12.70
Height (mm)	1.21	1.15	0.70	1.00	1.09	1.36
H/D	0.092	0.087	0.054	0.076	0.083	0.107

Table S2 The total shrinkage percentage of Ni-BZCYYb cermet anode and pure Ni anode during the reduction process within 8% and 100% H_2 concentration

	Ni metal		Ni-BZCYYb Cermet	
	100% H_2	8% H_2 + 92% N_2	100% H_2	8% H_2 + 92% N_2
Initial Diameter (mm)	13.34	13.26	12.87	12.87
Diameter after reduction (mm)	12.72	13.28	12.90	12.91
Percentage of shrinkage	4.65%	-0.15%	-0.23%	-0.31%
Porosity	26%	34%	42%	41%

Table S3 Electrochemical performance of NH_3 / air-fed PCFC

Anode	Electrolyte	Durability (h)	Ref.
Ni/CGO*	BCGO*	N/A	[51]
Pd	BZCY*	3 (@ 600 ^{o}C 0.6 A cm^2)	[48]
Ni/BZCYYbPd*	BZCYYbPd	130 (@ 500 ^{o}C 0.2 A cm^2)	[32]
Fe-decorared Ni-BZCYYb	BZCYYb	100 (@ 600 ^{o}C 0.5 A cm^2)	[52]
Fe-layered Ni-BZCYYb	BZCYYb	20 (@ 650 ^{o}C 0.5 A cm^2)	[53]
Pd-infiltrated NiO-BZCYYb	BZCYYb	100 (@ 500 ^{o}C 0.3 A cm^2)	[54]
Ni-BZCYYb4411*	BZCYYb4411	200 (@ 500 ^{o}C 0.7 V)	[55]
Ni | Ni-BZCYYb	BZCYYb	300 (@ 600 ^{o}C 0.35 A cm^2)	This work

*CGO: $Ce_{0.8}Gd_{0.2}O_{1.9}$, BCGO: $BaCe_{0.8}Gd_{0.2}O_{3-\delta}$, BZCY: $BaZr_{0.1}Ce_{0.7}Y_{0.2}O_{3-\delta}$,

BZCYYbPd: $Ba(Zr_{0.1}Ce_{0.7}Y_{0.1}Yb_{0.1})_{0.95}Pd_{0.05}O_{3-\delta}$, BZCYYb4411: $BaZr_{0.4}Ce_{0.1}Y_{0.1}Yb_{0.1}O_{3-\delta}$
